# Using the Ball-in-Bowl Metaphor to Outline an Integrative Framework for Understanding Dysregulated Emotion

**DOI:** 10.3389/fpsyt.2021.626698

**Published:** 2021-08-09

**Authors:** Ulrike Nowak, Martin F. Wittkamp, Annika Clamor, Tania M. Lincoln

**Affiliations:** Clinical Psychology and Psychotherapy, Institute of Psychology, Faculty of Psychology and Movement Sciences, Universität Hamburg, Hamburg, Germany

**Keywords:** affect regulation, emotion beliefs, psychophysiology, affective disturbances, affect dynamics

## Abstract

Dysregulated emotion plays an important role for mental health problems. To elucidate the underlying mechanisms, researchers have focused on the domains of strategy-based emotion regulation, psychophysiological self-regulation, emotion evaluations, and resulting emotion dynamics. So far, these four domains have been looked at in relative isolation from each other, and their reciprocal influences and interactive effects have seldom been considered. This domain-specific focus constrains the progress the field is able to make. Here, we aim to pave the way towards more cross-domain, integrative research focused on understanding the raised reciprocal influences and interactive effects of strategy-based emotion-regulation, psychophysiological self-regulation, emotion evaluations, and emotion dynamics. To this aim, we first summarize for each of these domains the most influential theoretical models, the research questions they have stimulated, and their strengths and weaknesses for research and clinical practice. We then introduce the metaphor of a ball in a bowl that we use as a basis for outlining an integrative framework of dysregulated emotion. We illustrate how such a framework can inspire new research on the reciprocal influences and interactions between the different domains of dysregulated emotion and how it can help to theoretically explain a broader array of findings, such as the high levels of negative affect in clinical populations that have not been fully accounted for by deficits in strategy-based emotion regulation and the positive long-term consequences of accepting and tolerating emotions. Finally, we show how it can facilitate individualized emotion regulation interventions that are tailored to the specific regulatory impairments of the individual patient.

## Introduction

Mental health problems are characterized by aberrant emotional experiences. These can take on many forms, such as low and flattened mood in the absence of context-sensitive emotional fluctuations in depression, chronically elevated levels and sudden peaks of anxiety in anxiety disorders, and emotional instability with sudden shifts between different emotional states in borderline personality disorder. These aberrant emotional experiences can be described best *via* aberrancies in emotion dynamics, that is, in the patterns with which emotional experiences fluctuate over time (1). It is likely that these aberrancies in emotion dynamics result from dysregulation in the domains of strategy-based emotion regulation (2), psychophysiological self-regulation (3), and emotion evaluations (4). Within each of these domains, research has focused on elucidating the underlying mechanisms in order to better understand the precise difficulties that drive mental health problems.

In the first part of this article, we briefly summarize the most influential models for each of these domain-specific literatures. We begin with the domain of strategy-based emotion regulation, which has been central to most of the clinical research on emotion regulation so far. We continue with the domains of psychophysiological self-regulation and emotion evaluations and finally summarize the research on emotion dynamics, which result from the other domains and thereby represent the outcome of the processes involved in strategy-based emotion regulation, psychophysiological self-regulation, and emotion evaluations. For each of the domain-specific literatures, we identify difficulties that their models have in explaining some robust empirical findings and discuss their limited usefulness for clinical practice. We then argue that the field could make more progress by moving beyond these domain-specific research foci, because they have hindered the understanding of reciprocal influences between the different domains of dysregulated emotion. We demonstrate how first attempts to connect some of these domains have been fruitful and how this potential could be optimized by an overarching framework that integrates all of the domains.^1^

In the second part of this article, we introduce a metaphor of a ball in a bowl that inspired our way of thinking about the interplay between the domains of dysregulated emotion. We explain how we adapted the metaphor for our purposes and then map the different metaphor elements on the four domains of dysregulated emotion. Finally, we show how the resulting outline of an integrative framework of dysregulated emotion can be used to delineate new research questions, can provide more comprehensive explanations for available research findings, and can be used to derive multimodal individualized interventions.

## Domains of Dysregulated Emotion

### Strategy-Based Emotion Regulation

Individuals can influence the intensity and duration of their emotional states *via* many different strategies. Although these processes are often simply referred to as emotion regulation (5), here we use the term “strategy-based emotion regulation” to differentiate these processes from other emotion regulatory mechanisms, such as psychophysiological self-regulation. The so far most influential account of strategy-based emotion regulation is the process model by Gross (6–8). In its earlier version, it introduced five categories of emotion regulation strategies. These include situation selection, situation modification, attentional deployment, cognitive change, and response modulation, each of which is suggested to take effect at a different temporal stage of the emotion generation process (6). Together, these categories of emotion regulation strategies are thought to constitute a strategy repertoire comparable to a toolbox from which the right tool needs to be selected and successfully applied to fix the problem at hand [for an explicit use of the toolbox terminology, see (9)]. The subsequently developed extended process model maps out three stages of emotion regulation, namely, (I) identifying the need to regulate, (II) selecting an appropriate strategy from the repertoire of emotion regulation strategies, and (III) implementing the selected strategy to modify the emotional state (7, 8).^2^ The extended process model postulates that emotion generation and the three emotion regulation stages can be broken down into a series of interlocking valuation cycles consisting of sequences of an aspect of the world, its perception, its valuation as positive or negative, and a resulting action. The extended process model postulates that deficits can arise at each subcomponent of each valuation cycle and that impairments in different components result in different symptoms of psychopathology (12, 13).

There have also been other extensions to the strategy-based model by Gross. One is the automatic emotion regulation account by Mauss et al. (14–16) that takes into account that emotion regulation strategies are not always consciously or willfully chosen but that their use can become automatic. Other emotion regulation theorists have pointed out that people regulate not only their own emotions but also those of other people [so-called social or interpersonal emotion regulation, see (17, 18)].

The strategy-based models of emotion regulation have stimulated an extensive research literature focusing on the frequency and success with which various strategies are employed. Specifically, researchers have looked at how the self-reported habitual use of different strategies is linked to emotional consequences and psychopathology cross-sectionally (2) and also prospectively (19). Experimental research has evaluated the effectiveness of different strategies to achieve short-term down-regulation of emotional states in experimental paradigms (20). Furthermore, experience-sampling studies have shed light on the frequency and short-term effectiveness of emotion regulation strategies in daily life [e.g., (21, 22)]. Researchers have also focused on flexibility and context sensitivity in strategy use (10, 23), automatic emotion regulation (15), and social emotion regulation (24). However, stages and components beyond the implementation stage have only recently started to attract some research attention [e.g., (9, 25)].

The strategy-based emotion regulation models have significantly advanced emotion regulation research. However, they also face explanatory difficulties. Their conceptual problems result in part from their grounding in the principles of cybernetic control theory (7, 26), an influential model of self-regulating systems applied in a broad range of contexts [e.g., (27–29)]. Key principles of control theory include comparisons of actual against desired states and the stipulation that discrepancies entail the application of counter-regulatory mechanisms. Building on these principles, the strategy-based models of emotion regulation are based on the implicit assumption that to prevent unhealthy affect, undesirable emotional states need to be reduced *via* the selection and implementation of modificatory strategies (12).^3^ Because of this assumption, the models are conceptually unable to account for robust evidence demonstrating that strategies such as awareness, acceptance, and tolerance, which do not involve reductions in the discrepancies between current and desired states (31, 32), nonetheless lead to positive mental health outcomes (22, 33, 34). Given this explanatory gap, it is not surprising that presentations of these models make no reference to such non-modificatory strategies as important components of the strategy repertoire (7, 13, 16, 17).

In contrast, the strategy-based emotion regulation account of Berking is not grounded in control theory principles and explicitly acknowledges the adaptive value of non-modificatory strategies [Adaptive Coping with Emotions (ACE) model; (31)]. The ACE model conceptualizes adaptive emotion regulation as a sequence of becoming aware of and labeling an emotion, analyzing its cause, providing self-support, and deciding to either modify or to accept and tolerate it. Contrary to the process model, the ACE model assumes both modificatory and non-modificatory strategies to be adaptive ways of dealing with emotional states. However, like the process model, it does not explain the mechanisms through which non-modificatory strategies confer their beneficial effects.

Second, strategy-based emotion regulation models have been criticized for their implicit assumption that emotional activation would perpetuate indefinitely unless emotion regulation strategies are applied: Kappas (35) points out that emotions are inherently self-regulating (I) because they motivate behaviors that tend to terminate the emotion-eliciting situation and (II) because they are governed by psychophysiological self-regulatory mechanisms that, following their activation, automatically activate processes to initiate their down-regulation [cf. (36, 37)]. The same point has also been highlighted by theorists who emphasize that experiencing emotions has beneficial effects that are facilitated by the self-regulatory characteristics of emotional experiences (38, 39). From this perspective, it is problematic that the process model attributes the aberrant levels of negative emotion that are reliably found in clinical populations [e.g., (40, 41)] solely to impairments in strategy-based emotion regulation (12, 13). Whereas questionnaire-based studies have reliably found deficits in strategy-based emotion regulation across mental disorders [for meta-analyses, see (42–45)], experimental studies have not consistently found the expected deficits in strategy use in clinical groups (46–49). Evidence from experience-sampling studies has also provided an inconclusive picture so far (50–52). This indicates that processes other than strategy-based emotion regulation need to be considered to account for the high levels of negative affect across clinical populations.

A third problem for the strategy-based emotion regulation models arises from evidence indicating that short- and long-term consequences of various emotion regulation strategies diverge. Specifically, maladaptive strategies such as suppression have been found to produce short-term reductions of undesired emotional states but negative longer-term emotional consequences (53). Conversely, adaptive non-modificatory strategies such as acceptance are less effective at achieving immediate emotional relief but show longer-term benefits for emotional states (54–57). As the strategy-based emotion regulation models define immediate reductions of the intensity of undesired emotions as regulation success (due to their being grounded in cybernetic control theory), they conceptually disregard the value of longer-term emotional consequences and imply that short-term beneficial effects also lead to longer-term emotional benefits. As a result, the strategy-based emotion regulation models do not specify which mechanisms account for discrepancies in short- and longer-term effects of different emotion regulation strategies.

Finally, Hofmann (58) noted that the direct contributions of strategy-based emotion regulation models to clinical practice have been limited. Some emotion regulation programs, such as emotion regulation therapy and the unified protocol, use the process model for psychoeducative purposes and encourage patients to enhance their repertoire of emotion regulation strategies (59–62). Also, Sheppes et al. (13) have argued that existing interventions, such as attentional bias modification (63), emotion regulation therapy (60, 61), dialectical behavioral therapy (64), and the affect regulation training (31), can be mapped onto different components of the extended process model. However, these programs were not directly derived from the process model and take a broader approach to emotion regulation including experiential, acceptance-based, and mindfulness-based techniques. Regarding the latter, the incompatibility of control-based models of emotion regulation with experience-based interventions has been highlighted (39), and Frederickson et al. (38) have argued that, in some situations, cognitive control strategies may even be detrimental as they can foster emotion avoidance.

In summary, strategy-based emotion regulation models offer a fine-grained account of subcomponents that are stipulated to constitute the emotion regulation process and have inspired a large research literature. However, because of their exclusive focus on strategy-based emotion regulation, they face explanatory difficulties to account for some of the findings in clinical emotion regulation research, and their usefulness for clinical practice is limited.

### Psychophysiological Self-Regulation

Emotions are, by nature, self-regulatory because of their underlying psychophysiological mechanisms [cf. (35, 39)]. When external and internal challenges are encountered, physiological processes initiate regulation, with the goal to achieve adaptation and return to homeostasis when possible (36, 37). An influential theoretical account of psychophysiological self-regulation that underlies many of the more specific models in this field is the allostatic load model by McEwen (37). The term allostasis literally means “stability through change.” It is used to refer to psychophysiological adaptation and suggested to be subserved by so-called allostatic systems including neuroendocrine systems such as the hypothalamus–pituitary–adrenal axis, the autonomous nervous system, and the immune system. According to the allostatic load model, the short-term activation of these allostatic systems prepares individuals to deal with challenges. Chronic activation, by contrast, constitutes allostatic (over)load that in the long term leads to wear and tear of the body and brain (65, 66). The model postulates that to allow allostatic systems to recover and to maintain their functionality, they include negative feedback loops that automatically induce down-regulatory effects and keep their activity quantity- and time-limited (36). However, these self-regulatory mechanisms can become dysregulated and impaired because of genetic and environmental factors (67), which results in individual differences in how well psychophysiological activation can be adapted in accordance with environmental demands.

To better understand mental health problems, more specialized theories of psychophysiological self-regulation are useful, such as the model of neurovisceral integration by Thayer and Lane (68). This model suggests that a neural network (the central autonomic network) regulates the heart *via* sympathetic (stellate ganglia) and parasympathetic outputs (vagus nerve). The model postulates that impairments in the rapid parasympathetic regulation of cardiac activity (indexed by low heart rate variability) compromise the ability of the organism to flexibly adapt to changing external or internal demands. In other words, the impairments reduce the psychophysiological self-regulatory capacity, which has a negative impact on emotional experiences and mental health (3). Similarly, the polyvagal theory by Porges (69) proposes that aberrancies in the vagal structures that regulate the heart and the resulting lack of psychophysiological self-regulation play an important role in psychopathology.

The allostatic load model and the vagal theories that followed from it have motivated numerous empirical studies. For example, investigators have delineated how different allostatic systems respond to emotional states (36) and how these systems are interrelated (70, 71). Further questions have referred to how genetic and environmental factors influence the development of psychophysiological self-regulatory capacity (72–74) and how psychophysiological self-regulation may be impaired differently in different clinical populations (3, 75). Furthermore, research stimulated by the model of neurovisceral integration has sought to trace the relationship between parasympathetic cardiac control and cognitive performance (76–78).

The vagal theories by Thayer and Lane (68) and by Porges (69) can be used to theoretically integrate psychophysiological self-regulation with strategy-based emotion regulation, based on the suggestion that these two domains are subserved by shared neuronal networks. Disinhibition in these shared networks may therefore be responsible for impairments in both psychophysiological self-regulation and in strategy-based emotion regulation (79–81). In support of this claim, meta-analytic evidence points to positive but small associations between strategy-based emotion regulation on the one hand and heart rate variability as an autonomic nervous system marker of psychophysiological self-regulation on the other (82, 83). In a different attempt to theoretically integrate psychophysiological self-regulation with strategy-based emotion regulation, Grecucci et al. (39) differentiated between top-down regulatory processes corresponding to strategy-based emotion regulation and automatic bottom-up processes corresponding to self-regulatory processes and further elaborated on how these routes are differentially addressed in psychological interventions.

The psychophysiological theories point towards mechanisms underlying psychophysiological self-regulation that can be addressed *via* a number of effective psychophysiological interventions when talking therapies are not sufficient [for a recent overview, see (84)]. For example, Mather and Thayer (80) reviewed how heart rate variability biofeedback stimulates vagal pathways and can positively influence functional connectivity in brain networks important for self-regulation. Recent empirical evidence corroborates this [e.g., (85, 86)]. Unlike the Gross model, the existing vagal theories made an effort to integrate strategy-based emotion regulation with psychophysiological self-regulation. However, although they are specific in the way they spell out the physiological underpinnings, they remain comparatively vague with regard to the specifications of strategy-based emotion regulation. For example, they do not elaborate on the different mechanisms *via* which modificatory and non-modificatory strategies are selected and how they affect emotional experiences. In addition, they do not integrate the domain of emotion evaluations, which plays a key role for dysregulated emotion.

### Emotion Evaluations

Some authors have postulated that how emotional states are experienced depends on the individual's evaluations of these emotional states. This idea is reflected in models about emotional schemas (87), implicit theories of emotion (88), attitudes toward emotions (89), and beliefs about emotions (90), which share the assumption that emotions are evaluated based on certain beliefs about emotions. The same idea also features in a recent model of metaemotions, in which emotion evaluations are theorized to represent necessary prerequisites for the development of metaemotions (91). In all of these models, evaluations of emotions can refer to many different aspects or attributes of emotional states, but these can be grouped into two central types of emotion evaluations, namely, (I) whether the emotional state is evaluated as helpful vs. harmful (which influences how motivated the individual is to alter the emotional state) and (II) whether it is evaluated as controllable or uncontrollable [i.e., whether the individual feels capable of changing the emotional state; (90)].

This conception stands in the tradition of early theories on how individuals evaluate external environmental demands. In their transactional process model, Lazarus and Folkman (92) postulate that individuals evaluate (I) the motivational relevance of an environmental demand, that is, whether it is potentially harmful. Furthermore, Lazarus and Folkman postulate that individuals evaluate (II) their coping capacities to overcome the presenting environmental demand [cf. controllability; (90)]. Both types of evaluations are assumed to mutually influence each other so that following an evaluation as potentially harmful, individuals' evaluations of their coping capacities further differentiate challenging from threatening perceptions of environmental demands (92, 93). In analogy to the Lazarus account, both an evaluation of an emotional state as potentially harmful and evaluations of personal capacities to deal with an emotional state can be assumed to crucially impact the further processing of this emotional state.

Although there has been relatively little research in this domain, empirical studies have started to test the assumptions stipulated in emotion evaluation theories. The focus of these studies has been on evaluations regarding the harmfulness and controllability of emotional states and how these are associated with emotional experiences, psychopathology, and well-being (88–90, 94–97). Furthermore, researchers have examined whether emotion evaluations can be changed and thus whether they are promising targets for psychological interventions (98).

Some progress has already been made in theoretically integrating emotion evaluations with strategy-based emotion regulation. Leahy (99) subsumed emotion evaluations and emotion regulation strategies under so-called emotional schemas, such as non-acceptance and rumination. Ford and Gross (90) added an evaluation component to the extended process model of emotion regulation (8). They suggest that beliefs regarding the harmfulness and controllability of emotional states motivate efforts to regulate these and influence the selection and implementation of regulatory strategies. In line with these considerations, several recent studies have focused on the idea that beliefs regarding the controllability of emotional states are associated with more active strategy-based emotion regulation efforts (4). In addition, associations between the evaluation of emotional states as unacceptable and the use of maladaptive regulatory strategies have been investigated (100).

A critique of the existing models of emotion evaluations is that most of them predominantly focus on evaluations concerning the controllability of emotional states (4), although the coping framework by Lazarus and Folkman (92) suggests a wider repertoire of evaluations to be relevant, including the capacity to accept. Also, despite having been inspired by Lazarus' original account, researchers have so far not investigated the interplay between evaluations of the harmfulness of emotional states and evaluations of personal capacities to deal with these emotional states (i.e., to change or accept them). This is a limitation because individuals' evaluations of an emotional state as potentially harmful and their evaluations regarding their ability to either modify or accept it can be expected to interact in influencing whether emotions are perceived as a manageable challenge or as an imminent threat and thereby influence which regulatory strategy will be used.

The failure to consider the interplay between harmfulness and capacity evaluations also limits the clinical utility of the existing evaluation models because it leaves limited potential for diagnosing the specific basis of individual problems in emotion processing (evaluations of the potential harmfulness of emotional states vs. evaluation of personal capacities to deal with emotional states vs. both). Consequently, there is also limited potential for designing and selecting specific interventions. Finally, the existing evaluation theories have their main focus on beneficial effects of high controllability evaluations. In line with control theory, they implicate that discrepancies between experienced and desired emotional states can be volitionally reduced at any time. This strong emphasis on controllability implies that people can always be in control of their emotional states which seems to be a problematic message to communicate to patients. As emotions often cannot instantaneously be controlled, supporting patients to tolerate their emotions may be equally relevant to enhancing mental health and well-being.

Taken together, there is a growing body of research on emotion evaluations with a predominant focus on beliefs regarding the controllability of emotional states, whereas beliefs regarding one's resources to accept and tolerate emotional states have received little attention. A consistent application of Lazarus' model to the emotion evaluation literature would help to remedy this problem and to gain insight into the interactions between harmfulness evaluations and evaluations of personal capacities to deal with emotional states. Finally, a better understanding of how emotion evaluations are linked with strategy-based emotion regulation efforts, psychophysiological self-regulation, and resulting emotion dynamics is still lacking.

### Emotion Dynamics

The term emotion dynamics refers to the patterns with which emotional experiences continuously fluctuate over time and thus reflects the outcome of the regulation effected by strategy-based emotion regulation, psychophysiological self-regulation, and emotion evaluations. Emotion dynamics have attracted the interest of many emotion theorists. To mention a few, Solomon and Corbit (101) described a normative sequence of how affect dynamically changes in response to environmental stimuli. In a first attempt to quantify affect dynamics, Richard Davidson (102) coined the term affective chronometry to describe the temporally dynamic features of emotional experiences and suggested defining features of emotion dynamics, such as rise time to peak and duration [for a more recent account, see (103)]. The seminal work of Frijda on the laws of emotion (104) distinguished different intensity profiles of emotion episodes. Most recently, Kuppens and Verduyn (105) put forward four principles that shape emotion dynamics, namely, the principles of contingency, inertia, regulation, and interaction.

Despite the extensive theorization on emotion dynamics, attempts to study them empirically have remained relatively scarce. The existing empirical studies have mostly focused on finding ways to capture individual differences in emotion dynamics. For this purpose, a number of arithmetically derived descriptors have been put forward. One is “emotional instability,” which represents the amount of frequent and extreme moment-to-moment fluctuations in emotion intensity and which is usually calculated from squared differences between emotion intensities at successive measurement points (106). Others are “emotional inertia”, which describes how strongly emotion intensities carry over from one moment to the next and is derived from autoregressive coefficients, and “emotional variability”, which describes the amplitude or range of affective fluctuations and is captured *via* the standard deviation (107). Further descriptors include “pulse”, reflecting variability in intensity, and “spin”, representing variability of qualitatively different emotional states in the two-dimensional core affect space of valence and arousal (108).^4^

These arithmetic descriptors have stimulated research that focuses on their associations with mental health outcomes. For instance, their application to experience-sampling data has been used to corroborate emotional instability in borderline personality disorder (113) and has also served as an inroad to delineating emotion dynamics in other mental disorders, including major depression, eating disorders, post-traumatic stress disorder, and psychosis (114–118). In a comprehensive meta-analysis, various forms of mental health problems were associated with more variable, unstable, but also more inert emotion dynamics (114). Aberrancies in emotion dynamics have also been analyzed as predictors of the transitions between episodes of psychopathology and mental health (1, 119, 120).

The literature of emotion dynamics still lacks a theoretical account that spells out the psychological or psychophysiological regulatory processes that influence emotional fluctuations over time. Nonetheless, there have been some empirical studies testing for associations between emotion dynamics and psychophysiological self-regulation as well as strategy-based emotion regulation. One found that emotional instability was linked with heart rate variability but not with strategy-based emotion regulation (121). Findings from others indicate that the use of reappraisal but not of suppression and rumination is associated with less inert emotional fluctuations over time (110, 112, 122, 123).

However, the lack of a theoretical model that specifies how emotion dynamics are shaped by different emotion regulatory processes renders empirical investigations in this field somewhat exploratory. The existing empirical findings cannot be interpreted in the context of an overarching framework, which prevents the generation of further-going research questions and theory-building. Also, the literature on emotion dynamics has not yet matured to a point where it directly benefits clinical practice. To reach this goal, disorder-specific aberrancies in emotion dynamics need to be reliably mapped and underlying mechanisms identified. Research activity could then be directed at developing interventions that ameliorate specific patterns of aberrant emotion dynamics.

### Interim Conclusions

For each domain of dysregulated emotion, we have now provided a brief summary of the most influential theoretical models, the research questions they have stimulated, and their strengths and weaknesses for research and clinical practice. We conclude that strategy-based models would benefit from taking into account the self-regulatory functions of psychophysiological systems to be able to more explicitly incorporate experiential and acceptance-based approaches. In turn, psychophysiological accounts would benefit from being integrated with the specifications of how different emotion strategies are selected and how they affect emotional experiences. Research on emotion evaluations would benefit from incorporating psychophysiological self-regulation, which would move — besides controllability evaluations — the benefits of evaluations of resources to accept and tolerate into focus. Finally, more research is needed to explore how emotion dynamics are shaped by strategy-based emotion regulation, psychophysiological self-regulation, and emotion evaluations. In summary, we conclude that a fully overarching framework that allows an integration of all four domains would advance the progress the field is able to make. Such an integrative framework would have to be able to explain how different aspects of dysregulated emotion act and interact leading to mental health problems, to account for findings that have so far remained conceptually unexplained, and would need to facilitate the tailoring of intervention approaches to individual patients' needs. In the following, we will describe how we outlined such an integrative framework inspired by the metaphor of a ball in a bowl, which we used as a crutch to help us to integrate the different domains of dysregulated emotion.

## The Ball-In-Bowl Metaphor

### The Original Ball-In-Bowl Metaphor by Boker

Boker uses the metaphor of a ball placed in a bowl to develop a framework for psychological systems that include multiple regulatory forces (124). His recourse to a metaphor stands in the tradition of many influential scientific models that have benefited from metaphorical thinking, such as the tree of life in Darwinian conceptualizations of evolution (125), references to waves and particles in physics (126, 127), and metaphorical conceptualizations of attention as a moving spotlight or a limited resource in cognitive psychology and neuroscience (128). Psychological interventions also employ metaphors to facilitate understanding, such as when mindfulness is compared to watching clouds moving across the sky (129). The use of metaphors has thus proven helpful for conceptualizing complex processes in theory and clinical practice.

The concept of emotion regulation itself has also already been approached *via* analogies. In the context of the Gross model, the repertoire of emotion regulation strategies has been compared to a toolbox from which the right tool has to be selected and successfully applied (9). Furthermore, and with some resemblance to the metaphor we will be presenting here, Grecucci et al. (130) illustrated how emotion regulatory processes are initiated in the brain by using an analogy from statistical mechanics, a branch of physics that applies probability theory to thermodynamic systems. They propose that, similar to the thermodynamic regulatory processes captured by the Boltzmann distribution, the brain initiates regulatory mechanisms — which in this model take on the form of psychodynamic defenses — when the tolerability threshold to bear the respective emotion is exceeded.

In Boker's ball-in-bowl metaphor, the bottom of the bowl represents the preferred equilibrium point of the ball. When an external force operates on the ball, the ball is set in motion and moves up the bowl walls. At some point, the imposed external force and the force of gravity are in balance, and the ball maintains a stable position. When the external force terminates, the ball finds its way back to its preferred equilibrium at the bottom of the bowl, due to the force of gravity and the curvature of the bowl. Boker considers this to be a fast regulation process resulting from an automatic balancing of forces. He also assumes that if the external force continues to apply over an extended period of time, the ball remains at its new, non-preferred equilibrium point up the wall of the bowl. However, in such situations, the bowl can be tilted (i.e., leaned sideways) in such a way that the ball is again situated at the bottom of the bowl. Importantly, the bowl is now no longer in a level position but tilted to the side. Boker calls this process adaptation and conceptualizes it at a slower timescale than the fast regulation process resulting from the curvature of the bowl.

From the metaphor of a ball in a bowl, Boker derives his Adaptive Equilibrium Framework (124). This framework offers a multiprocess regulation account that is meant to inform models of various self-regulating human systems. In the application of his framework to emotion regulation, Boker takes the ball to represent either positive or negative emotion. However, he does not specify the psychological processes corresponding to the stipulated fast regulation process (=bowl curvature) and the slower adaptation process (=tilting the bowl). Therefore, Boker does not connect his framework to the domains of strategy-based emotion regulation, psychophysiological self-regulation, and emotion evaluations.

### Adapting the Ball-In-Bowl Metaphor

We started with the simple ball-in-bowl metaphor by Boker and then adapted and extended it in a way that would make it possible to map the specific metaphor elements onto the domains of strategy-based emotion regulation, psychophysiological self-regulation, emotion evaluations, and emotion dynamics. We remained with Boker's image of a ball in a bowl to represent how emotions (=the ball) are constrained by two distinct regulatory forces (=bowl curvature and bowl-tilting). However, we amended Boker's account in how the effects of bowl-tilting are specified. We also added varying degrees of bowl curvature and colored zones in the bowl walls. In this section, we will present the individual elements of our adapted ball-in-bowl metaphor, staying within the language of the metaphor. In the following section, we will map each metaphor element onto one of our psychological concepts of interest, namely, strategy-based emotion regulation, psychophysiological self-regulation, emotion evaluations, and emotion dynamics.

#### Dynamic Ball Movements

**Figure 1:**
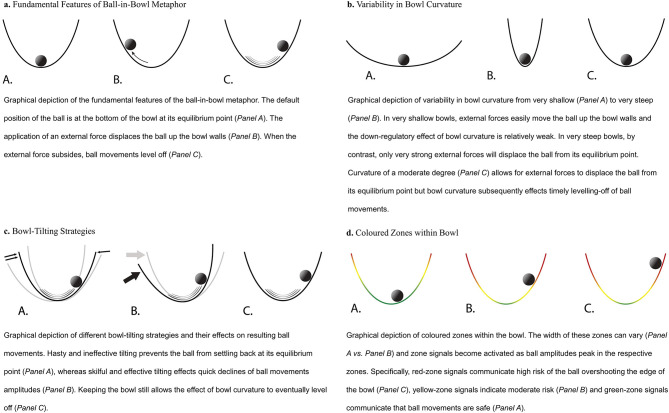
Graphical depictions of the different elements of the bowl-in-bowl metaphor. **(a)** Fundamental Features of Ball-in-Bowl Metaphor. **(b)** Variability in Bowl Curvature. **(c)** Bowl-Tilting Strategies. **(d)** Colored Zones Within Bowl.

Like Boker's metaphor, the adapted metaphor posits a ball in a bowl with its equilibrium point at the bottom of the bowl (Figure 1a, Panel A). When an external force is applied, it drives the ball up the bowl walls (Figure 1a, Panel B). When the external force is terminated or reduced, the trajectory of the ball eventually levels off at the bottom of the bowl (Figure 1a, Panel C).

#### Bowl Curvature

Both Boker's account and the adapted metaphor explain the leveling-off of ball movements *via* bowl curvature, which draws the ball back toward the bottom of the bowl. However, the adapted ball-in-bowl metaphor introduces the additional feature that bowl curvature can differ on a continuum from very shallow to very steep. In shallow bowls, the effect of bowl curvature is relatively weak. As a result, when an external force is applied in shallow bowls (Figure 1b, Panel A), the ball easily moves up the walls and subsequently also takes longer until its movements level off. By contrast, in very steep, vase-like bowls, external forces will rarely be strong enough to effect ball movements (Figure 1b, Panel B). Finally, in bowls with a moderate degree of curvature (Figure 1b, Panel C), external forces are assumed to lead to some dynamic ball movements but these subside in a timely manner.

#### Bowl-Tilting Strategies

Alongside the effect of bowl curvature, both Boker and the adapted ball-in-bowl metaphor stipulate that the bowl can be tilted (i.e., leaned sideways) to influence the trajectory of the ball. Unlike Boker's account, however, the adapted metaphor specifies that this tilting can either be hasty and ineffective (Figure 1c, Panel A), which prevents ball movements from leveling off, or skillful and effective (Figure 1c, Panel B), which facilitates the settling down of ball movements. Finally, the adapted metaphor explicitly includes the possibility of keeping the bowl stable to leave it to bowl curvature to bring the ball back to its equilibrium point (Figure 1c, Panel C).

#### Colored Zones

In the adapted ball-in-bowl metaphor, the bowl walls are divided into three zones colored green, yellow, and red. These signal the extent to which ball movement amplitudes peaking in these zones bear the risk of overshooting the edge of the bowl. Green-zone signals communicate that ball movement amplitudes are safe (Figure 1d, Panel A), yellow-zone signals give an indication of moderate risk (Figure 1d, Panel B), and red-zone signals communicate high risk (Figure 1d, Panel C). The width of these zones can vary, and this determines the specific thresholds of ball movement amplitudes at which green-, yellow-, and red-zone signals are elicited (Figure 1d, Panels A,B).

In summary, the adapted ball-in-bowl metaphor includes three regulatory processes that influence how the ball dynamically moves within the bowl. These are (I) bowl curvature (Figure 1b), (II) bowl-tilting skills (Figure 1c), and (III) the colored zones that signal risk of ball movement amplitudes overshooting the edge of the bowl (Figure 1d). Furthermore, the adapted metaphor specifies that variation is possible for each of these regulatory processes so that bowl curvature can differ in steepness, tilting capacity in aptness, and colored zones in their widths.

### Mapping the Metaphor Elements Onto the Domains of Dysregulated Emotion

Contrary to Boker, who did not specify the psychological analogs of his metaphor elements, we then mapped all components of the adapted ball-in-bowl metaphor onto the corresponding domains of dysregulated emotion (for an overview, see Table 1).

**Table 1:** Elements of the ball-in-bowl metaphor and their psychological analogs.

**Elements of the ball-in-bowl metaphor**	**Psychological analogs**
Ball	Emotion
Amplitudes of ball movements	Emotion intensities
Fluctuations in ball movement amplitudes	Emotion dynamics
Bowl curvature	Psychophysiological self-regulatory capacity
Bowl-tilting	Strategy-based emotion regulation
Colored zones	Emotion evaluations

#### Ball Movements as Emotion Dynamics

In the adapted ball-in-bowl metaphor, the ball represents discrete emotions such as anxiety or anger. External forces that set the ball in motion stand for internal or external events through which the emotion becomes activated. When the ball lies at its equilibrium point at the bottom of the bowl, this corresponds to a state in which the emotion is at its zero point. The adapted metaphor also specifies that the amplitude of the ball movements corresponds to emotion intensity, and alterations of ball amplitudes over time represent temporal emotion dynamics.

#### Bowl Curvature as Psychophysiological Self-Regulatory Capacity

Different degrees of bowl curvature (shallow–steep) represent individual differences in psychophysiological self-regulation capacity. More shallow bowls correspond to reduced capacity for psychophysiological down-regulation, which is associated with faster and higher increases in emotion intensity and longer recovery times. By contrast, very steep, vase-like bowls that allow for no ball movement represent high rigidity. Like low psychophysiological self-regulation capacity, this can be assumed to be maladaptive because emotions convey important information, and some emotional responsiveness is needed to engage with environmental challenges.

#### Bowl-Tilting as Strategy-Based Emotion Regulation

Within the adapted metaphor, bowl-tilting represents the application of emotion regulation strategies. Targeted and skillful tilting corresponds to adaptive modificatory emotion regulation strategies, such as reappraisal. These strategies are considered adaptive because when properly applied they successfully remove momentum from emotion dynamics.^5^ In addition, keeping the bowl still until ball movements have leveled off corresponds to non-modificatory strategies, such as awareness, acceptance, and tolerance. These constitute a second type of adaptive strategies as over time they also effect reductions in emotional momentum. By contrast, hasty and ineffective tilting that keeps the ball in motion corresponds to maladaptive emotion regulation strategies, such as rumination and suppression. These are referred to as maladaptive strategies because either they do not reduce emotional intensity at all, or they hastily achieve emotional down-regulation, but without actually reducing the emotional momentum. This results in renewed emotion peaks and long-term emotional instability, which in turn initiates continuous regulatory efforts.

#### Colored Zones as Emotion Evaluations

The colored zones on the bowl walls represent evaluations of whether the intensity of emotional states bears the potential for harm. Specifically, ball movement amplitudes in the red zone, which signal high risk for the ball to overshoot the edge of the bowl, correspond to evaluations of emotion intensities as threatening. Ball movement amplitudes in the yellow zone, which signal moderate risk of ball movement amplitudes, correspond to evaluating emotion intensity as challenging but manageable. Finally, ball movement amplitudes in the green zone, communicating that ball movements are safe, correspond to emotion intensities evaluated as helpful.

These colored zones and the corresponding signals of threat (red zone), challenge (yellow zone), and helpfulness (green zone) result from the combination of harmfulness evaluations and evaluations of personal capacities to modify or accept emotional states. Harmfulness evaluations refer to the motivational relevance, that is, whether they are helpful or bear the potential for harm. Evaluations of personal capacities to deal with the emotional state include modificatory strategies such as cognitive reappraisal and/or non-modificatory strategies such as awareness, acceptance, and tolerance. Hence, when an emotional state is evaluated as potentially becoming harmful, an evaluation of individual abilities to deal with the emotional state determines whether it is evaluated as a manageable challenge (yellow zone) or as a threat (red zone). Only when resources to either modify or to accept and tolerate the potentially harmful emotional state are evaluated as insufficient, it will be evaluated as threatening.

### Using the Adapted Metaphor to Outline an Integrative Framework of Dysregulated Emotion

The idea at the heart of this article is that dysregulated emotion plays an important role for mental health problems and can be described best *via* aberrancies in emotion dynamics that arise due to dysregulation in one or more of the domains of strategy-based emotion regulation, psychophysiological self-regulation, and emotion evaluations. In clinical research, empirical studies on the interactive effects between these domains have been relatively scarce, at least in part because an integrative framework that can guide such cross-domain investigations has been lacking. We found that the adapted ball-in-bowl metaphor can be a helpful crutch to outline such an integrative framework, firstly because it facilitates an intuitive understanding of the complex cross-domain interactions and secondly because it prepares the ground for theory-guided research based on clear and directed hypotheses and thereby prevents haphazard correlational studies that lack sufficient theoretical underpinning.

In a nutshell, the adapted ball-in-bowl metaphor suggests that aberrant ball movements (i.e., dysregulated emotion) can arise because of (I) deficient bowl-tilting skills (i.e., maladaptive strategy-based emotion regulation), (II) shallow or overly steep bowl curvature (i.e., maladaptive psychophysiological self-regulation), and (III) overly prominent red zones (i.e., dysfunctional evaluations of emotional states). These three domains are assumed to influence each other in their impact on the resulting ball movements (i.e., emotion dynamics). Multiple interactive effects can be derived from the adapted ball-in-bowl metaphor. For example, shallow bowls come with less down-regulation of ball movements, but good tilting skills can compensate for this and can help to achieve reductions in ball movement amplitudes. Furthermore, red-zone signals motivate hasty tilting attempts to prevent the ball from overshooting the edge of the bowl.

**Figure 2:**
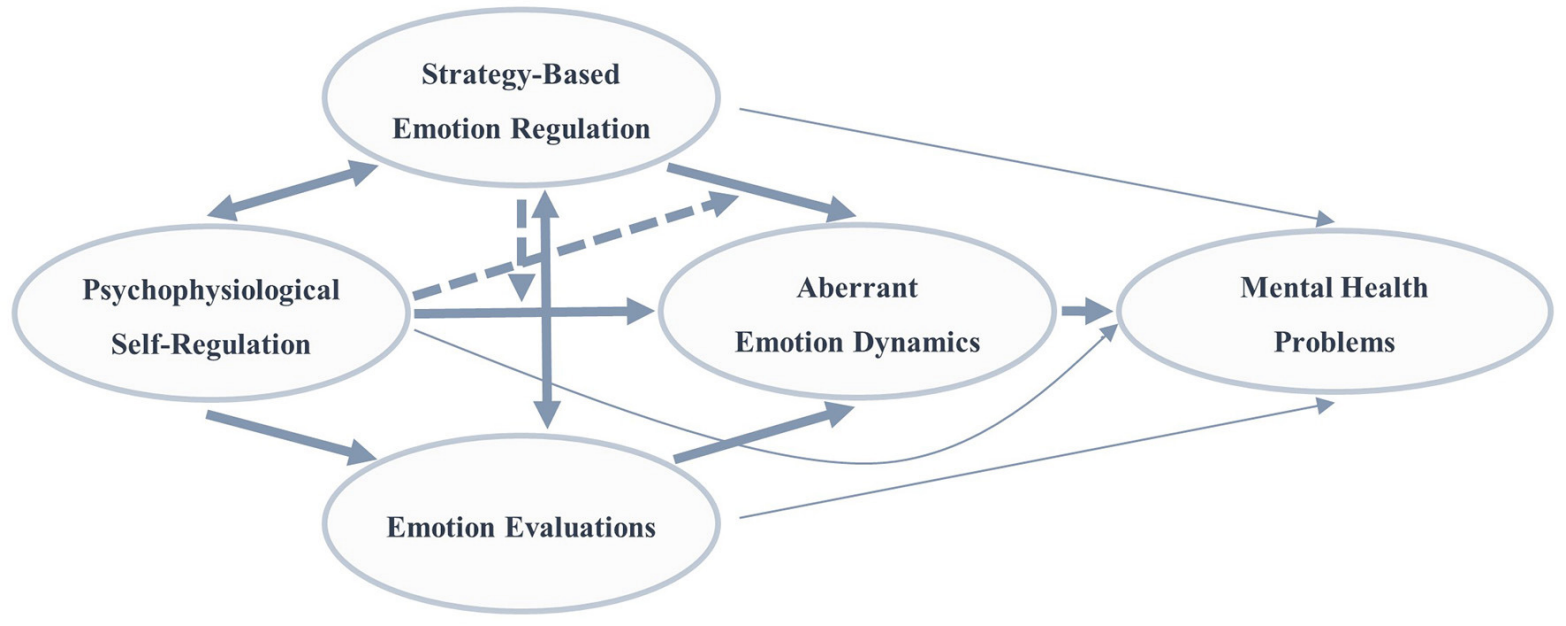
Graphical illustration of the integrative framework of dysregulated emotion. Emotional dysregulation is assumed to occur as a result of the interplay between strategy-based emotion regulation, psychophysiological self-regulation, and emotion evaluations and to be reflected in aberrant emotion dynamics. Mental health problems are expected to result from dysregulated emotion. Hypothesized paths are indicated by arrows. Solid bold arrows depict direct associations or mediations *via* another domain. Dotted arrows illustrate moderation effects. Besides their main path *via* aberrant emotion dynamics, all domains are also assumed to be directly associated with mental health problems, represented here *via* thin arrows.

These metaphor-based specifications can thus be used to outline a framework for understanding dysregulated emotion (Figure 2) and, more importantly, can be translated into a series of testable hypotheses on how the interplay of aberrancies in the four domains of strategy-based emotion regulation, psychophysiological self-regulation, emotion evaluations, and resulting emotion dynamics may result in mental health problems. Specifically, the assumptions outlined in the previous paragraph, formulated within the language of the metaphor, can be translated into the corresponding psychological terms, which provides the outline of a new dysregulated emotion framework. Strategy-based emotion regulation and psychophysiological self-regulation moderate their respective effects on emotion dynamics. Specifically, low psychophysiological self-regulatory capacity is likely to make strategy-based emotion regulation more challenging. In reverse, adaptive strategy-based emotion regulation could compensate for low psychophysiological self-regulatory capacity or a rigid psychophysiological response to the environment. Furthermore, deficits in one domain can be assumed to produce cascading effects in the other domains, thereby potentiating the effects on resulting emotion dynamics. Specifically, threatening evaluations of emotional states could be associated with frequent use of maladaptive emotion regulation strategies such as rumination or suppression. This is then likely to be linked with aberrant emotion dynamics and with continuous regulatory efforts. By contrast, evaluations of emotional states as challenging but manageable can be hypothesized to be associated with adaptive strategies, such as reappraisal and acceptance. These are assumed to effectively reduce emotion intensities, thereby preventing renewed emotional peaks and aberrant emotion dynamics. Finally, low psychophysiological self-regulation and poor strategy-based emotion regulation can be assumed to give rise to repeated experiences of sustained emotional activation in the absence of adequate resources to deal with these emotional states. This is likely to foster evaluations of emotional states as threatening.

Furthermore, the integrative framework of dysregulated emotion suggests that emotion dynamics represent the central pathway from dysregulation in strategy-based emotion regulation, psychophysiological self-regulation, or emotion evaluations to mental health problems. Thus, we expect that in most situations, dysregulation in strategy-based emotion regulation, psychophysiological self-regulation, or emotion evaluations will distort emotion dynamics and that mental health problems then follow from these distortions. Nonetheless, the framework also allows for direct and isolated influences from strategy-based emotion regulation, psychophysiological self-regulation, and emotion evaluations to mental health problems (thin arrows in Figure 2). Hence, the framework does not preclude the possibility that dysregulation in a single domain has a direct influence on mental health problems regardless of the emotion dynamics.

### Integrating Existing Findings

The proposed links of the integrative framework of dysregulated emotion are backed up by a range of existing findings, such as the well-replicated observation that heart rate variability and other indicators of psychophysiological self-regulation are associated with strategy-based emotion regulation (82, 83) and with aberrant emotion dynamics (121), by the links between strategy-based emotion regulation and aberrant emotion dynamics [e.g., (110, 123)], and by the associations between evaluations of emotions and the use of different types of emotion regulation strategies (4, 96). Furthermore, the framework is in line with research indicating robust associations between aberrant emotion dynamics and mental health problems (114). In addition, it can explain some robust findings that the existing models summarized in the first part of this article have struggled to make sense of. One is that non-modificatory strategies, such as awareness, acceptance, and tolerance, which do not in and of themselves achieve reductions in emotion intensities, have nonetheless repeatedly been shown to predict positive mental health outcomes (22, 34). The integrative framework suggests that non-modificatory strategies do not directly modify emotional states but that they enable the psychophysiological self-regulatory processes to do so. Finally, by focusing only on one or two aspects of emotion regulation at a time, the existing models of emotion regulation have struggled to fully account for the high levels of negative affect in clinical populations. In contrast, the integrative framework suggests that high levels of negative affect can occur due to dysregulation located either in strategy-based emotion regulation, or in psychophysiological self-regulation, or in emotion evaluations. Thus, while one domain, such as strategy-based emotion regulation, may be intact, high levels of negative affect may result from problems in other domains.

### Clinical Usefulness

A major advantage of the adapted ball-in-bowl metaphor is that it can be used in clinical interventions as a simple and intuitive, transdiagnostic explanatory model that stimulates individualized approaches to emotion dysregulation. The metaphor encourages practitioners to not prematurely assume that regulation difficulties must be strategy-based in nature, but to also consider psychophysiological self-regulation, emotion evaluations, and their joint effects on resulting emotion dynamics. In the mindset of the integrative framework of dysregulated emotion, emotion regulation interventions would start with individualized case formulations that profile each patient's impairments and resources in the framework domains. From this analysis, patient-specific intervention plans can be derived. Depending on patients' profiles, these plans may or may not include work on strategy-based emotion regulation (31, 131–133), psychophysiological self-regulation [e.g., *via* relaxation techniques, physical exercise, or heart rate variability biofeedback training; see (134–136)], and emotion evaluations [e.g., *via* techniques from cognitive therapy; see (137)]. Such individualized interventions are likely to be more effective than the one-size-fits-all approach of currently available emotion regulation trainings.^6^ Furthermore, ecological momentary interventions (139) may be promising to further advance individualized interventions as they could be programmed to detect dysregulated emotion and to prompt personalized supportive input.

Second, the adapted ball-in-bowl metaphor can serve as an easily accessible visualization tool for the direct work with patients because it can illustrate specific emotion regulation problems and their consequences. For example, the metaphor can be used to explain why maladaptive strategies such as rumination and suppression keep emotional activation going, why emotional states do not necessarily require modificatory regulation to decline, and how evaluations of emotions as threatening lead to hasty and ineffective strategy-based regulation attempts that aggravate rather than resolve the emotional difficulties at hand. Furthermore, the flexibility of the metaphor with regard to which specific emotion is represented by the ball makes it a versatile tool that can be applied to the specific emotion regulation problems of individual patients.

### Limitations and Future Directions

The adapted ball-in-bowl metaphor and the integrative framework of dysregulated emotion are not without constraints. First, they build on the distinction between adaptive and maladaptive strategies. This categorization has been criticized and adaptive emotion regulation has been said to involve the flexible selection of strategies in accordance with situational demands (10, 23). Although we agree that rigid classifications of individual strategies are problematic, we nonetheless justify this distinction with the robust research evidence showing that the habitual use of certain strategies is associated with psychopathology (2). Another potentially problematic aspect is that we conceptualized maladaptive strategies as capable of achieving short-term reductions of emotion intensity. Alternatively, it may be the case that the short-term benefit of maladaptive strategies lies not primarily in emotion intensity reductions but more in the subjectively experienced sense of control that accompanies using these strategies [for a similar line of argument, see (140)]. Furthermore, it is also conceivable that the different domains could have been mapped onto other than the allocated elements of the metaphor. However, the chosen pairings seemed the most intuitive ones and have proven advantageous for deriving compelling hypotheses. In addition, it must be noted that the metaphor of a ball in a bowl is just one of many conceivable metaphors to aid the integration of the domains of dysregulated emotion. Other metaphors might emphasize different interrelations between the network domains, which would result in different model formulations.

Looking ahead, the integrative framework of dysregulated emotion awaits empirical testing and the clinical utility of the metaphor needs to be evaluated in direct work with patients. Future work could also focus on extending the framework to include additional domains. For instance, it may be helpful to add emotional awareness as a separate domain, represented in the metaphor as the detection, identification, and non-judgmental monitoring of a specific kind of ball and the amplitude of its movements. Similarly, compassionate self-support could be included as an additional domain that could be represented by a confident attitude regarding bowl-tilting [(141); also cf. compassion-focused therapy, (142)]. Another metaphor extension could introduce the possibility that more than one ball can simultaneously be in the bowl. This would represent multiple concurrently activated emotions that may also interact with each other. Moreover, it is conceivable that more than one strategy is applied to deal with an emotion [cf. (143)] and that multiple maladaptive strategies could be used in a hasty attempt to reduce emotion intensity.

In addition, research should focus on the genetic and environmental factors that influence the developmental pathways of the framework domains, especially during sensitive developmental periods. For instance, observational learning in childhood can be assumed to influence the development of strategy-based regulation (144); childhood adversities, and inflammations of the immune system are likely to constitute risk factors for decreased psychophysiological self-regulation capacity (67, 145), and caregivers' beliefs about emotions and their reactions to emotion displays may shape individuals' emotion evaluations.

In summary, the integrative framework of dysregulated emotion derived from the adapted ball-in-bowl metaphor can inspire a new generation of theory-driven research activity with the potential to explain a broader array of research findings and to increase the usefulness of the field for clinical practice.

## Data Availability Statement

The original contributions presented in the study are included in the article/supplementary material, further inquiries can be directed to the corresponding author.

## Author Contributions

UN, MW, and TL developed the idea. UN and MW wrote the first draft of the manuscript. TL provided extensive feedback and made amendments. AC provided advice on specific sections and feedback on the final version of the manuscript. All authors have approved the final manuscript.

## Conflict of Interest

The authors declare that the research was conducted in the absence of any commercial or financial relationships that could be construed as a potential conflict of interest.

## Publisher's Note

All claims expressed in this article are solely those of the authors and do not necessarily represent those of their affiliated organizations, or those of the publisher, the editors and the reviewers. Any product that may be evaluated in this article, or claim that may be made by its manufacturer, is not guaranteed or endorsed by the publisher.

## Footnotes

^1^Readers who are familiar with the research on the domains of strategy-based emotion regulation, psychophysiological self-regulation, emotion evaluations, and emotion dynamics are invited to skip our brief review of these literatures and to directly move to the second part of this article.

^2^For similar three-staged accounts of emotion regulation, see Bonanno and Burton (10) and Webb et al. (11).

^3^For further control theory accounts of emotion regulation, see Larsen (30) and Webb et al. (11).

^4^For mathematically more advanced methods of capturing emotion dynamics, see Chow et al. (109); Kuppens et al. (110); Montpetit et al. (111); Oravecz et al. (112).

^5^Here, the adaptiveness of emotion regulation strategies refers to their effectiveness to down-regulate emotional states, under the assumption that the present emotional state is appraised as challenging or threatening. However, when an emotional state is appraised as helpful, up-regulation of emotion intensity can also be adaptive. The reader is referred to the subsequent paragraph in which we discuss how emotion evaluations are represented in the adapted ball-in-bowl metaphor.

^6^For a similar argument regarding interventions for negative symptoms in schizophrenia, see Lincoln et al. (138).
